# Supplementary material for: Reconciling Mining with the Conservation of Cave Biodiversity: A Quantitative Baseline to Help Establish Conservation Priorities
Source: PLoS One. 2016 Dec 20;11(12):e0168348. doi: 10.1371/journal.pone.0168348 (PMC5173368; doi:10.1371/journal.pone.0168348)
Supplement: S1 Dataset — (ZIP) [file pone.0168348.s002.zip › Taxa/Serra Norte/SN_2013/N4WS/Lista N4WS-26.pdf]

CAVIDADE N4WS-0026

Classe	Ordem	Fam/Outros	Gên/Outros	Espécie	S-U	U-S	Zon.
Arachnida	Acari	Bdellidae		sp.2		X	E
Arachnida	Acari	Galumnidae		sp.1	X		E
Arachnida	Acari	Galumnidae		sp.2	X		E
Arachnida	Acari	Veigaiidae		sp.1	X		E
Arachnida	Amblypygi	Charinidae	*Charinus*	jovem		X	E
Arachnida	Araneae	Dipluridae		jovem		1	E
Arachnida	Araneae	Ochyroceratidae	*Ochyrocera*	sp.1		X	E
Arachnida	Araneae	Ochyroceratidae	*Ochyrocera*	sp.2	X		E
Arachnida	Araneae	Oonopidae	aff. *Matiotia*	sp.1		X	E
Arachnida	Araneae	Oonopidae	aff. *Triaeris*	sp.1		X	E
Arachnida	Araneae	Pholcidae	*Mesabolivar*	*eberhard*	X	X	E
Arachnida	Araneae	Salticidae	*Freya*	sp.1	X		E
Arachnida	Araneae	Theraphosidae		jovem		3	E
Arachnida	Araneae	Theridiidae	*Achaearanea*	sp.1		X	E
Arachnida	Araneae	Theridiidae	*Episinus*	sp.3	X		E
Arachnida	Araneae	Theridiosomatidae	*Plato*	sp.1	X	X	E
Arachnida	Pseudoscorpiones	Chthoniidae	*Pseudochthonius*	sp.1	X	X	E
Entognatha	Collembola	Entomobryidae		sp.6		X	E
Entognatha	Collembola	Paronellidae		sp.1	X		E
Insecta	Coleoptera	Carabidae		jovem	X		E
Insecta	Coleoptera	Staphylinidae	Pselaphinae	sp.8	X		E
Insecta	Diptera	Canacidae		jovem		X	E
Insecta	Diptera	Phoridae	Phorinae	sp.	X		E
Insecta	Diptera	Psychodidae	*Sciopemyia*	*sordellii*		X	E
Insecta	Diptera	Tipulidae	Tipulinae	sp.	X		E
Insecta	Heteroptera	Schizopteridae	Schizopterinae	jovem	X		E
Insecta	Hymenoptera	Formicidae	*Camponotus*	sp.1	X		E
Insecta	Hymenoptera	Formicidae	*Hypoponera*	sp.1	X		E
Insecta	Hymenoptera	Formicidae	*Nylanderia*	sp.2	X		E
Insecta	Hymenoptera	Formicidae	*Nylanderia*	sp.3		X	E
Insecta	Hymenoptera	Formicidae	*Pachycondyla*	*striata*		1	E
Insecta	Hymenoptera	Formicidae	*Solenopsis*	sp.1	X		E
Insecta	Isoptera	Termitidae	*Nasutitermes*	sp.	X	X	E
Insecta	Lepidoptera	Noctuoidea		sp.1	6	12	E
Insecta	Lepidoptera	Noctuoidea		sp.2		X	E
Insecta	Orthoptera	Phalangopsidae	*Paraclodes*	sp.	6	2	E
Insecta	Psocoptera	Psyllipsocidae	*Psyllipsocus*	sp.3		X	E
Insecta	Psocoptera	Ptiloneuridae	*Triplocania*	sp.1	X	X	E
Insecta	Thysanoptera	Thripidae		sp.1		X	E
Malacostraca	Isopoda	Philosciidae		sp.1		X	E
Amphibia	Anura	Strabomantidae	*Pristimantis*	cf. *fenestratus*	1		E
Amphibia	Anura			sp.1	1		E
